# An exploration of wellbeing in men diagnosed with prostate cancer undergoing active surveillance: a qualitative study

**DOI:** 10.1007/s00520-022-06976-w

**Published:** 2022-03-19

**Authors:** Omar Eymech, Oliver Brunckhorst, Louis Fox, Anam Jawaid, Mieke Van Hemelrijck, Robert Stewart, Prokar Dasgupta, Kamran Ahmed

**Affiliations:** 1 MRC Centre for Transplantation, Guy’s Hospital Campus, King’s College London, King’s Health Partners, London, UK; 2 Translational Oncology and Urology Research (TOUR), School of Cancer and Pharmaceutical Sciences, King’s College London, London, UK; 3 Institute of Psychiatry, Psychology and Neuroscience, King’s College London, London, UK; 4 South London and Maudsley NHS Foundation Trust, London, UK; 5 Urology Centre, Guy’s and St, Thomas’ NHS Foundation Trust, King’s Health Partners London, London, UK; 6 Department of Urology, King’s College Hospital, London, UK; 7 Department of Urology, Sheikh Khalifa Medical City, Abu Dhabi, United Arab Emirates

**Keywords:** Active surveillance, Mental health, Mental wellbeing, Prostate cancer, PSA anxiety, Quality of life

## Abstract

**Purpose:**

There is a growing emphasis on improving quality of life of people with prostate cancer. However, those undergoing active surveillance remain underrepresented in the literature with less known about their unique challenges. Therefore, we aimed to explore their lived experiences post diagnosis and its effect on their mental, social, and physical wellbeing.

**Methods:**

Qualitative semi-structured interviews were conducted with 13 men undergoing active surveillance for low-risk disease. Thematic analysis was used to inductively co-construct themes through the lens of the biopsychosocial model.

**Results:**

Mental wellbeing was strongly affected in our participants due to the overwhelming emotional impact of their diagnosis resulting in an ‘Emotional Diagnostic Disequilibrium’. Informational awareness and education about prostate cancer helped patients with ‘Recognition of the Impact’. Patients experienced an ‘Unsettling Monitoring Cycle’ due to the increased fear and anxiety around PSA monitoring appointments, with some men ignoring their mental wellbeing needs as their disease is ‘A Future Problem’. ‘Concealment of Diagnosis’ left many feeling isolated and highlighted an important coping mechanisms in the ‘Importance of a Social Support Network’ theme. Finally, physical health mostly changed through alterations in health behaviour, leading to ‘A Healthier Lifestyle’ with increasing attribution of physical symptoms to age through ‘Symptomatic Overshadowing’.

**Conclusion:**

The greatest disease impact on men’s wellbeing was at the time of diagnosis, with a subsequent cyclical anxiety and fear of disease progression prominent around monitoring appointments. Future research should explore ways to better support patients with these issues and at these times, improving their quality of life.

**Supplementary Information:**

The online version contains supplementary material available at 10.1007/s00520-022-06976-w.

## Introduction

As the most common male cancer in Europe, prostate cancer remains an important public health problem [1]. With globally growing incidences and improving survival, the number of men who are living with and beyond disease is increasing. Therefore, there is a growing realisation of the impact prostate cancer has on the physical, mental and social wellbeing of men. The physical effects of disease and treatment remain well documented [2], but increasingly the mental impact of a diagnosis is being acknowledged. Anxiety and depression are prevalent in this group, with other aspects of mental wellbeing such as sense of masculinity, altered body image perceptions and low self-esteem commonly encountered [3, 4]. Additionally, social relationships, particularly those with the intimate partner, are also impacted [5]. Therefore, when considering factors impacting the quality of life of cancer patients, it is important that a holistic approach is taken to consider individual’s various health dimensions. Engel’s biopsychosocial model, which was further refined over the years [6], provides a systematic way of considering the following constructs: physical, mental and social wellbeing [7]. Additionally, the World Health Organization’s (WHO) International Classification of Functioning, Disability and Health Framework (ICF) provides a standard for recording, framing, describing and measuring functioning and disability in patients, which can be relevant to those receiving cancer care [8].

Prostate cancer is however a disease of wide varying spectrum when considering the ranging natural history, severity and treatments undergone. For men diagnosed with low-risk disease (Gleason score of 6, prostate-specific antigen < 10 ng/ml and stages T1-2), active surveillance is widely considered the treatment of choice [9]. This consists of close observation for disease progression in patients through a structured surveillance programme consisting of regular prostate specific antigen (PSA) testing and clinical examination along with MRI imaging and repeat biopsies where required. Curative treatment, such as surgery or radiotherapy, can then be offered if signs of progression are identified. The aims of active surveillance are to avoid overtreatment, and its associated toxicity, in men who do not yet, or will not require treatment without compromising overall survival [10]. However, the frequent lack of physical symptoms in these patients due to a lack of a radical intervention means the biopsychosocial model on its own is not sufficient to explain the impact of disease on wellbeing in these men. It is also important to consider prostate cancer as a diagnostic label and a major life event having an impact on individuals’ psychosocial health [11, 12]. In addition, the perception of living with untreated cancer, and the frequent uncertainty associated with active surveillance, are significant [13]. Despite this complex interaction between disease and wellbeing in this group, existing studies focus more on the quantitative evaluation of either physical or psychosocial factors rather than the holistic evaluation of the impact of disease [14] However, quantitative studies using questionnaires and functional assessment tools likely underestimate the impact, and unique challenges experienced during the active surveillance management option may not be adequately represented [15]. Furthermore, the few qualitative studies in this field focus more on the specific perception of living with untreated disease, rather than the biopsychosocial and holistic impact this has on patients [16]. Alternatively, studies evaluate the role of psychosocial factors in the selection of active surveillance as a treatment method, alongside barriers to adherence [17, 18]. There is therefore a need for further qualitative studies in this group, providing a holistic in-depth account of the experiences of men so that adequate support for this often-overlooked cohort can be provided. Therefore, we aimed to qualitatively explore the experiences of patients with prostate cancer undergoing active surveillance and describe the effect this has on their wellbeing.

## Methods

### Study design

This qualitative study was carried out following the Standards for Reporting Qualitative Research (SRQR) guidelines [19]. A phenomenological approach was utilised to elicit participants lived experiences of undergoing active surveillance for their prostate cancer diagnosis. We used an interpretive/constructivist paradigm for the analysis acknowledging individuals interpretation of the world around them and their experiences along with that of socially constructed knowledge [20]. These were underpinned by the previously mentioned biopsychosocial and major life event models when exploring and analysing the effect of undergoing active surveillance on men [6, 12]. Prospective ethical approval was granted by an NHS Research Ethics Committee (REC Reference: 20/SC/0070) with all participants providing informed, written consent.

### Participants

A purposive sampling strategy was used to recruit men from active surveillance clinics at a single tertiary unit in London, UK, who were undergoing active monitoring for their disease through PSA, imaging and biopsy when required. During screening of potential participants, we seeked participants with diverse backgrounds, disease duration and ages to ensure a wide representation of disease experience. Participants were eligible if they were over 18 years of age, had received a histologically proven prostate cancer diagnosis and had only received active surveillance as a management option. Those previously treated with surgery, radiotherapy, chemotherapy or any anti-androgen therapy were excluded. Patients were eligible regardless of ethnicity, civil status and sexual orientation. A total of 33 potential individuals were sent the patient information leaflet for participation in the study, out of which 15 consented to take part. Eight individuals did not consent due to time constraint, four believed prostate cancer had no effect on their wellbeing, three did not wish to have their responses recorded due to the sensitive nature of the topic and three provided no reason. Of the 15 consenting to participate, two dropped out for undisclosed personal reasons.

### Data collection

Semi-structured interviews were conducted from March to August 2021 via video-based online interviews to maintain participant safety due to the COVID-19 pandemic. Participants were informed during the screening call about the purpose of the study and the sensitive nature of the interview questions. Participants were given the option to conduct interviews alone or with someone else, e.g. a partner. All our participants, however, opted to conduct the interview on their own. A semi-structured format was chosen to enable consistency of topics covered across interviews whilst allowing for in-depth exploration of the participants’ wellbeing and flexibility in issues not directly related to the questions [21]. Interviews were conducted at a time convenient to the participant by one of two researchers: OE or OB (Bachelor and PhD male students respectively with clinical backgrounds, formal qualitative interviewing training and no prior relationship to participants). A topic guide was developed in line with a literature review and the biopsychosocial model, targeting the developed initial questions to elicit responses related to phenomenological experiences of individuals. This was piloted prior to use, and as we conducted more interviews, this was refined with questions added based on the analysis of the already collected data (Online Resource 1). Interviews were audio recorded with interviewer notes taken and participant characteristics collected. Data was subsequently pseudonymised and each participant given a study number identifier.

### Data analysis

Analysis started after the first interview, with audio interviews transcribed verbatim and exported onto NVivo 11 software to aid with coding management. We utilised an inductive thematic analysis conducted through the lens of the biopsychosocial model [22]. This was chosen to inductively generate new findings within widely acknowledged components described in the biopsychosocial model of health. Two researchers (OE and OB) co-constructed emergent themes through a process of data familiarisation, code generation and subsequent theme generation using a thematic map underneath biopsychosocial model construct headings of mental, social and physical wellbeing. Regular meetings were held throughout to discuss codes and themes generated and their context within the existing framework. Reflexivity was maintained by the research team through questioning style, maintenance of a reflexive diary and regular meetings to discuss findings against previous assumptions [23]. We sought to increase our analytical rigour through multiple coders, consensus meetings and the use of an audit log. We additionally utilised investigator triangulation with a research team consisting of varying levels of previous exposure to prostate cancer patients clinically. Our initial target sample was set at 15 participants based on our study aims, sample specificity and analysis methods; however, data saturation was reached after 13 participants as no new concepts emerged from the data [24]. All participant characteristics collected were presented through descriptive statistics.

## Results

### Participant characteristics

Thirteen participants underwent semi-structured interviews from a diverse range of ethnic backgrounds, ages, and disease duration (Table 1). Participant ages ranged from 57 to 74 (mean 66 years) and time since diagnosis varied between 1 and 7 years (mean 4 years). All but three participants were married (with an average time with partner of 31 years) and all identified as heterosexual. Six were (46%) Caucasian, four (31%) Black Caribbean, two (15%) Black African and one (8%) mixed White-Vietnamese. Seven interviewees were retired, and six were still in full/part-time work. Interview durations varied from 30 to 70 min with an average of 45 min.

**Table 1:** Detailed participant characteristics

Participant number	Age (years)	Time since diagnosis (years)	Civil status	Time with partner (years)	Occupation	Sexuality	Ethnicity
1	66	4	Married	25	Retired	Heterosexual	White
2	70	2	Married	44	Retired	Heterosexual	White
3	74	4	Married	49	Retired	Heterosexual	White
4	71	7	Married	41	Retired	Heterosexual	White
5	68	6	Married	45	Retired	Heterosexual	White
6	68	2	Married	26	Working	Heterosexual	Black African
7	57	5	Married	1	Working	Heterosexual	Black Caribbean
8	67	5	Single	N/A	Working	Heterosexual	Black Caribbean
9	60	2	Married	5	Working	Heterosexual	White/Vietnamese
10	66	4	Single	N/A	Retired	Heterosexual	Black Caribbean
11	60	4	Single	N/A	Working	Heterosexual	Black Caribbean
12	59	1	Married	30	Working	Heterosexual	White
13	67	3	Married	43	Retired	Heterosexual	Black African

### Findings

**Fig. 1:**
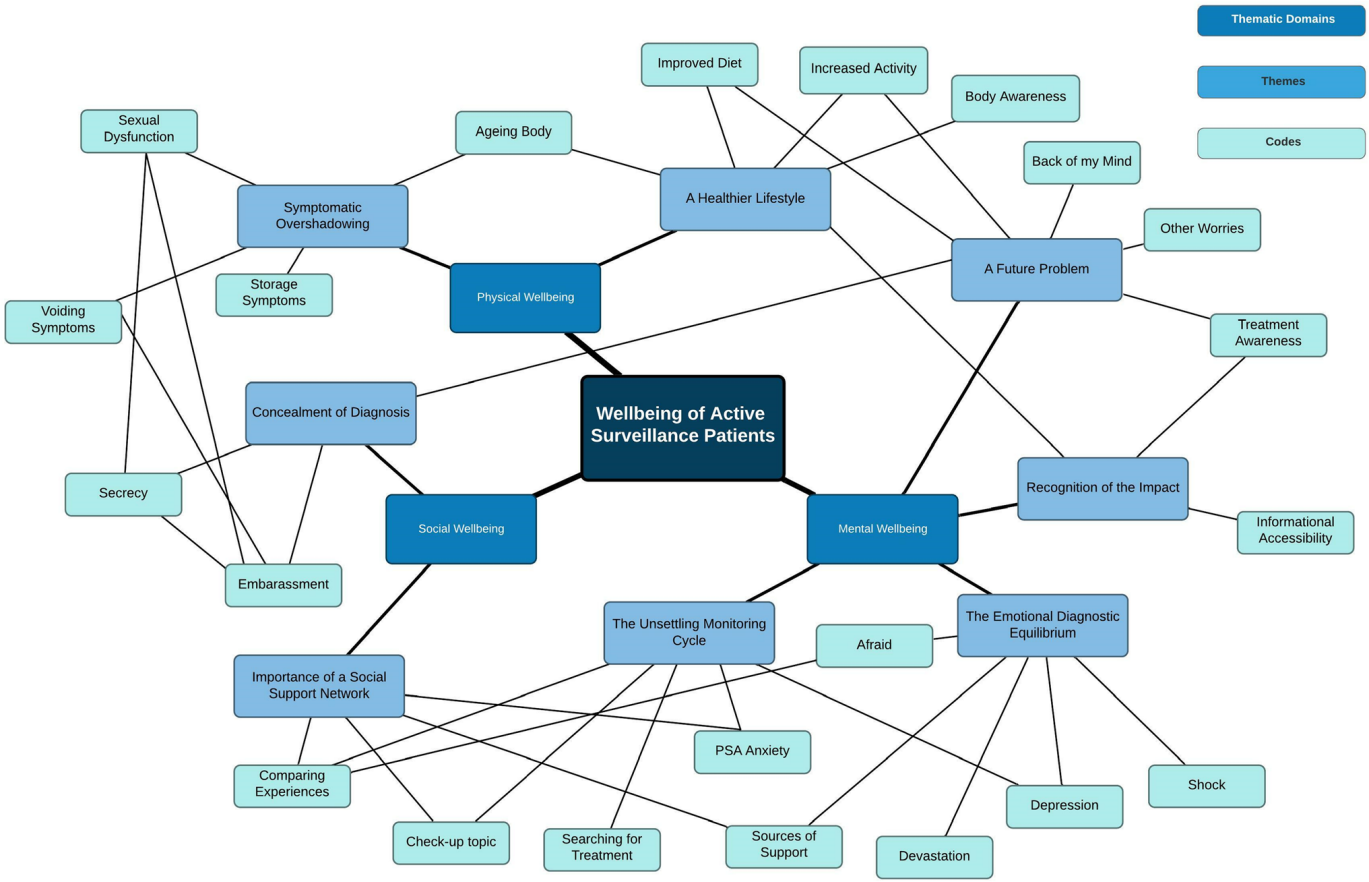
Mind map connecting generated themes and sub-themes within biopsychosocial framework utilised

Seventy-four codes (Online Resource 2) and 8 descriptive themes (Fig. 1) were generated under the predefined constructs of wellbeing according to the biopsychosocial model. Mental wellbeing was the most affected construct, accounting for most of the generated codes with themes generated including ‘The Emotional Diagnostic Disequilibrium’, ‘Recognition of the Impact’, ‘The Unsettling Monitoring Cycle’ and ‘A Future Problem’. Individuals’ social wellbeing generated two themes: ‘Concealment of Diagnosis’ and ‘Importance of Social Support Network’. Physical wellbeing was least coded with themes predominantly focussed on changes in health behaviour including ‘A Healthier Lifestyle’ and ‘Symptomatic Overshadowing’.

### Mental wellbeing

Themes generated described how participants were affected at different stages throughout their disease journey. Common patterns were seen in certain groups divided into those with little previous exposure to prostate cancer and those who had previous knowledge of the disease through family or friends affected by it.

#### The emotional diagnostic disequilibrium

The initial stages of disease had a clear impact on the mental wellbeing of participants. The entire diagnostic experience was an overwhelming experience. Commonly, anxiety, uncertainty, shock, disappointment and low mood were experienced, starting even before the diagnosis itself when the possibility of cancer was introduced by the healthcare professionals. There were differences however between those who were going through a routine PSA check and those who had previous symptoms, with the latter being less surprised and shocked at high PSA levels and subsequent need for further investigations.

The period immediately after receiving the cancer diagnosis presented the strongest emotional trigger during the diagnostic journey. This difficult time was filled with several emotions including shock, disbelief, surprise, disappointment and anxiousness at what lay ahead. Some clearly remembered the event itself and its impact although being several years ago, demonstrating its importance as a major life event despite being diagnosed with ‘low risk’ disease requiring no treatment. All of our participants described disappointment, with those who had little to no awareness of prostate cancer often more shocked and overwhelmed at the time: ‘*It was like in a film. I was like, Oh, my, God, the world just stopped still. It was like, I’ve got cancer, I can’t believe I’ve got cancer. There was that moment where I was just standing there in a sort of sheer disbelief that I had cancer. So, in that morning, it was almost like the world stood still for me. I just couldn’t believe that I got cancer’ (participant 2, White, age 70).*

#### Recognition of the impact

As men began to gather more knowledge about their disease, and as its threat became perceived as non-immediate, many described an easing to anxiety. Information seeking was common at this point, with an important element of this being the repeated appointments with healthcare professionals where further information was provided after the initial shock of diagnosis had subsided: ‘*Me personally, I associated with, you know, imminent death. But as it started to go on, and they started to explain to me and as time went on, and then more discussions with different doctors, I realised that if I’m monitored properly, then I can live as long as I would live anyway*.’ *(participant 11, Black Caribbean, age 60)*.

Many described the prospect of a low-grade disease was not as bad as initially believed, but still there was a recognition of what the diagnosis could potentially mean for their future lives. Many recognised that multiple aspects of their lives needed to change to accommodate the impact of their illness, with many acknowledging that they required an increase in support, particularly emotionally for their feeling of overwhelm, anxiety and low mood. This recognition was important, allowing men to acknowledge their illness and accept a sick role, in turn encouraging their willingness to seek the needed emotional help and allowed them to restore some sense of normality back to their lives.

#### The unsettling monitoring cycle

Following the recognition of the impact of the disease, a somewhat normal state of mind returned to many. Several described learning to manage to continue with their daily lives without worrying about their disease. During this time, they did not speak about or address their prostate cancer. This was however temporary, with a repeated reminder about their illness through an upcoming PSA test or follow-up appointment: *‘But I don’t have any symptoms, other than every six months, so I have to talk to someone about it.’ (participant 3, White, age 74)*. In the lead up to that period, some described a cyclical period of heightened ‘fear’ and ‘anxiety’, especially whilst waiting for the results of any tests taken: *‘The worst bit was the actual waiting for the results. This plays tricks on your mind’ (paticipant 12, White, age 59)*. Many became obsessed with their PSA levels, keeping meticulous records of their results for comparison with previous levels. This gave some a sense of control over their disease; however, this could quickly be lost through elevated results, triggering an emotional disequilibrium again. As a result, participants lived in a constant cycle of a relapsing–remitting anxiety related to the increased obsession with their PSA value around the time of appointments: ‘*I don’t worry about it on a day-to-day basis or month-to-month basis. Every six months when I go to my check-up appointment, I get worried about it, but I get informed of what’s happening. and then we move on*.’ *(participant 5, White, age 68)*.

#### A future problem

Due to the low-grade nature of their disease and the lack of a radical treatment, some participants did not address prostate cancer frequently, believing it was not something that concerned them at the time: ‘*I threw it under the carpet and have not dealt with it*’ *(participant 9, White/Vietnamese, age 60)*. However, many instead chose to label their cancer as ‘a future problem’ instead. There was an acknowledgement, particularly around those who had profuse knowledge and health literacy on the subject, that their disease may progress, with many fearing what lay ahead in terms of surgery or radiotherapy if that was the case. This demonstrated that despite labelling prostate cancer as a future problem, there was an ongoing impact on their current wellbeing through a persistent fear of progression of disease that remained constantly in the back of their minds: *‘It was always in the back of my mind*’ *(participant 11, Black Caribbean, age 60).*

### Social wellbeing

There were important implications for men and how they interacted with their partners, family and friends. Two contrasting views were expressed with some opting for isolation through concealment, and others instead acknowledging the importance of maintaining good social relationships as an invaluable source of support.

#### Concealment of diagnosis

Men would usually disclosed their diagnosis to their significant other. However, some decided to keep their diagnosis from other close family members or friends, commonly citing that they did not want to worry them, choosing to protect them from the news: ‘*I’m trying not to lay my problems on other people. And if I don’t have problems, then hopefully they’re not harbouring them’ (participant 5, White, age 68)*. Instead, many felt that due to the low-risk nature of their disease, it was not worth the trouble to themselves or particularly others to discuss their diagnosis until there was a more serious threat to their health: ‘*I know they would be very worried and panic. Unless it moves into a worse position, then I don’t really want to have that family discussion just yet’ (participant 9, White/Vietnamese, age 60)*. This concealment, however, came at the expense of their own wellbeing. Men described being left with little social support, fighting their own battles without family or friend support, meaning an increased feeling of loneliness was felt.

#### Importance of a social support network

Despite participants sometimes concealing their diagnosis from others, many acknowledged how important maintaining a good support network was. For many, the support of their partner was crucial throughout the process, with men describing how they would not be able to cope without them. Men therefore sympathised with those who could not rely on this source of support: *‘They all haven’t got a supportive wife; without her, it wouldn’t have been such an experience for me where I felt confident about going forward. I think men who are on their own will find it much more difficult than maybe men who are married.’ (participant 1, White, age 66)*. Secondly, many acknowledged the role of close friends, particularly when individuals may be going through comorbidity of their own due to their age. Additionally, other prostate cancer patients in the form of friends or even support groups were also a useful source of support as they felt *‘better understood’ (participant 13, Black African, age 67)* and that they could ‘*compare notes and experiences’ (participant 4, White, age 71)*.

### Physical wellbeing

Due to the lack of an active treatment for their cancer, physical complications and side effects of treatments did not burden our participant sample. Themes generated under this heading mainly described how men perceived their physical wellbeing to have been affected following cancer diagnosis in accordance to the ICF model (body function, activities and participation). The physical wellbeing of men was mostly altered through new health behaviours or further attributing existing problems to their age.

#### Symptomatic overshadowing

Many believed that problems which predated their diagnosis or even those that arose after were simply secondary to their age as opposed to being caused by their prostate. Fatigue, urinary symptoms and sexual dysfunction were commonly attributed to this: *‘I’m not expected to be going at it (referring to sex) at this old age’ (participant 5, White, age 68)*. However, other participants believed these symptoms could also be due to cancer but are being ‘overshadowed’ by old age and other comorbidities. Nevertheless, these physical problems frequently impacted other aspects of their lives and their social and mental wellbeing. This included affecting their relationships with their partner due to sexual dysfunction, reduced attendance at leisure activities because of fatigue and reduced social interactions secondary to the fear of embarrassment from an episode of urinary incontinence.

#### A healthier lifestyle

Interestingly, the cancer diagnosis led to many becoming more aware of their body and the issues that impacted their physical wellbeing. Men started to seek online information about risk factors and lifestyle modifications that can be helpful in their fight against cancer. This led to a positive change in many, referencing changes such as consuming less red meat or reducing alcohol intake. Several men increased their physical activity levels by going to the gym more, pursuing a new sport or simply by going on more walks. The reasoning for this change was often reported as a hope that it would slow down the growth of the cancer to avoid subsequent treatment: *‘I really don’t want to have any radiotherapy or chemotherapy or anything like that, if I can avoid it.’ (participant 12, White, age 59)*.

## Discussion

Issues pertaining to quality of life in prostate cancer have drawn increasing attention. However, patients undergoing different management options have widely differing experiences after being diagnosed. We provide an insight into the issues experienced by men undergoing active surveillance; an often underrepresented and overlooked group in the literature.

It is clear from our findings that despite the lack of an active treatment and being labelled with low-risk disease, a diagnosis of prostate cancer had important implications for participants. This demonstrates the often-underestimated nature of the impact of disease on this group which is seen in quantitative studies [15]. This was particularly the case for their mental and social wellbeing. As is common after any cancer diagnosis, shock and disbelief were a prominent feature in our participants initially [25]. However, more uniquely to active surveillance, once this subsided through an understanding of the true nature of the disease and recognition of its potential impact on daily life, a common and important cyclical anxiety was seen around appointment times, with distress and a newfound obsession with their PSA levels seen in many. Similarly, an ongoing fear of progression was always in the back of their minds. These findings are consistent with some of the previous quantitative literature available, which highlight high prevalences of ‘PSA Anxiety’ and ‘Fear of Cancer Recurrence’ amongst men undergoing active surveillance [26]. This further reinforces the need to address these linked domains of mental wellbeing during active surveillance follow-up. It is important to therefore monitor for these either through direct questioning or by using validated prostate cancer specific tools such as the Memorial Anxiety Scale for Prostate Cancer (MAX-PC) [27]. This allows for the identification of these problems early, meaning appropriate referral for treatment could be made. It has been demonstrated that interventions such as cognitive behavioural therapy (CBT) offer an effective means to improve these symptoms and thereby could improve the quality of life of active surveillance patients [28]. A combination of online and in-person CBT was demonstrated to be an effective method to reduce distress, also known as the 6th vital sign [29], in a sample of colorectal cancer patients [30]. Further research into the use of such hybrid interventions in prostate cancer patients is therefore important to consider due to their possible efficacy and cost-effectiveness.

The social impact of disease in our participants was also important. Many hid their diagnosis from close family, often at detriment to their own mental and social wellbeing. Several factors were hypothesised in the literature pertaining to the concealment of cancer diagnosis, such as cultural, religious and gender reasons. However, this remains an underresearched topic that requires further qualitative and observational studies to ascertain these reasons, and offer measures in place to shift patient behaviour [31]. The importance of a good social network and having men who have undergone similar experiences were seen to be important coping mechanisms for our participants. This has previously also been highlighted in other studies, describing men’s tendency to seek support from those with similar illness experiences [32]. These findings have important clinical implications, particularly when considering the evidence for peer support in improving depressive symptoms in prostate cancer patients [33]. Whilst support groups are commonly available as part of cancer services, few are targeted or suited to patients undergoing active surveillance. The unique experiences and issues encountered mean that patients would benefit from their availability as part of local or wider services to allow for the opening up of more specific problems encountered in this cohort [34].

Due to the low-grade nature of prostate cancer in our participant sample, specific cancer symptoms did not burden the majority of participants. Men wanted to improve their physical health to slow down disease progression and be better prepared to fight their cancer during its advanced stage. This complements the findings of a large cohort study which shows a transient improvement in physical health observed when comparing pre- and immediate post-diagnosis state [35].

A strength of our study is the high proportion of our sample drawn from patients of Black African and Caribbean ethnic backgrounds. African-Caribbean individuals have a high prostate cancer incidence and mortality, and it is therefore vital to capture their experiences and understand the issues that affect this patient population [36, 37]. Furthermore, our study recruited a wide range of ages of participants with a variety of disease duration to reflect a range of experiences. The study also has some limitations. Firstly, the biopsychosocial model received criticism in the literature for being outdated following the increased understanding of the biological underpinnings affecting various aspects of mental life, and hence making their separation unnecessary [38]. Although we based our theoretical foundation of this study on Henningsen’s refinement of the biopsychosocial model and the major life events theory [6, 12], other models such as WHO’s ICF could be considered more clinically relevant and have seen increasing use in psycho-oncology research as a result [8, 39]. Secondly, none of our participants identified as gay or bisexual, so it was not possible to explore the experiences of this group who have previously been identified to experience significant problems post-diagnosis with sexual identity and relationships [40]. Furthermore, most of our participants were married, meaning the views of single men were underrepresented with previous research suggesting that they may experience greater difficulty [41]. Lastly, due to the COVID-19 pandemic, all our interviews had to be conducted utilising video-based online platforms rather than face-to-face. Whilst increasingly being utilised and proven to be an effective data collection modality [42, 43], there is a possibility this could have affected rapport during interviews and selected against people with lower socio-economic backgrounds due to a lack of access to the required resources.

Future qualitative research in active surveillance patients should firstly target some of the underrepresented populations from this study and the wider literature such as single men and those identifying as gay or bisexual. However, importantly, based on our findings, future research should focus on addressing the issues encountered in our participants. There is a need to evaluate more targeted methods to improve the wellbeing and quality of life of this unique group of prostate cancer patients. The role of support groups for active surveillance patients requires further initial qualitative evaluation to establish its acceptability and need, followed by quantitative trials to determine its effectiveness. Similarly, there is a need to develop and establish the effectiveness of interventions to improve issues encountered during active surveillance particularly ongoing PSA anxiety and fear of disease progression. If effective, these would undoubtedly improve the lived experiences of patients who undergo active surveillance for prostate cancer.

## Conclusions

Prostate cancer patients undergoing active surveillance remain an underrepresented group within the literature when evaluating the effect of disease on men. We provide an insight into the lived experiences of these men, and the impact disease has on their mental, social and physical wellbeing. The impact was greatest around the time of diagnosis, with subsequent cyclical anxiety and fear of disease progression around PSA monitoring appointments. The importance of a good social network for support was also seen. Future research should explore ways to better support patients with these issues to improve the lived experiences of men undergoing active surveillance for prostate cancer.

Supplementary information.

## Supplementary Information

Below is the link to the electronic supplementary material.

Supplementary file1 (DOCX 16 KB)

Supplementary file2 (DOCX 18 KB)

## Data Availability

Anonymised data utilised for the analysis of this review is available to bona fide researchers following reasonable requests to the corresponding author.
