# Supplementary material for: An exploration of wellbeing in men diagnosed with prostate cancer undergoing active surveillance: a qualitative study
Source: Support Care Cancer. 2022 Mar 19;30(6):5459–68. doi: 10.1007/s00520-022-06976-w (PMC8933126; doi:10.1007/s00520-022-06976-w)
Supplement: Supplementary file 2 — Supplementary file2 (DOCX 18 KB) [file 520_2022_6976_MOESM2_ESM.docx]

**Online Resource 2 – List of Generated Codes During Data Analysis**

| Code | | |
| --- | --- | --- |
| **Physical Wellbeing** | | |
| Ageing Body | Inconvenience | Storage Symptoms |
| Body Awareness | Increased Activity | Use of Aids |
| Fatigue | Loss of Libido | Voiding Symptoms |
| Improved Diet | Sexual Dysfunction |  |
| **Mental Wellbeing** | | |
| Acceptance | Initial Difficulty | Searching for Treatment |
| Adjustment | Health over Dignity | Shock |
| Afraid | Informational Accessibility | Threat |
| Avoiding Dwelling | Loss of Interest | Treatment Awareness |
| Avoiding Support | Low Mood | Unaddressed |
| Back of my Mind | Need More to be Done | Unaware |
| Belittle the Impact | Not a Threat | Uncertainty |
| Check-up Topic | Other Worries | Unexpected |
| Control | Overwhelmed | Unusual |
| Delaying Surgery | Paranoid | Volatile |
| Depression | Previous Exposure | Wary of Threats |
| Devastation | PSA Anxiety | What Caused This? |
| Fighting Own Battles | Reassurance | Why Me? |
| Formality | Reflection |  |
| Future Problem | Reluctancy to Seek Help |  |
| **Social Wellbeing** | | |
| Advocate | Misunderstanding | Secrecy |
| Being there for My Family | Need to Know | Separation |
| Burden | Not Taken Seriously | Sources of Support |
| Comparing Experiences | Other Forms of Intimacy | Undesired Topic |
| Embarrassment | Precautions | Worried About Me |
| Exaggerate | Private Procedures | Wrong Time |
| Financial Stability | Pushed by my Wife |  |
